# Congenital Heart Diseases in Children: A Narrative Analysis of Early Diagnosis, Clinical Management, and Long-Term Outcomes

**DOI:** 10.7759/cureus.113213

**Published:** 2026-07-23

**Authors:** Mohamed Suhail Mohamed Nabi, Rashmi Ranjan Barik, Ankit Sharma, Garima, Chanchal Sharma, Venugopal Reddy I, Aithagoni Rajesh Goud

**Affiliations:** 1 Department of Computer Applications, The New College (Affiliated to the University of Madras), Chennai, IND; 2 Department of Paediatrics, Sriram Chandra Bhanja Medical College and Hospital, Utkal University, Cuttack, IND; 3 Department of Paediatrics, Subharti Medical College, Swami Vivekanand Subharti University, Meerut, IND; 4 Department of Obstetrics and Gynecology, Sanskriti Ayurvedic Medical College and Hospital, Mathura, IND; 5 Department of Ayurveda, Ayurvedic and Unani Tibbia College, New Delhi, IND; 6 Department of Paediatrics, Medicover Hospital, Bengaluru, IND; 7 Department of Clinical Sciences and Medical Education, Manipal Academy of Higher Education, Manipal, IND

**Keywords:** cardiac surgery, congenital heart disease, echocardiography, long-term outcomes, newborn screening

## Abstract

Congenital heart disease is one of the most common congenital anomalies in children and remains a major contributor to neonatal morbidity, infant mortality, repeated hospitalisation, and lifelong cardiovascular care needs. The clinical spectrum ranges from minor septal defects to complex cyanotic and duct-dependent lesions requiring urgent intervention. The objective of this review is to analyse early diagnosis, clinical management, and long-term outcomes of congenital heart diseases in paediatric populations, with attention to screening, multidisciplinary treatment, complications, and follow-up needs. A narrative literature search was conducted using PubMed, Scopus, Web of Science, and Google Scholar for articles published from 2015 to 2026. Eligible sources included original studies, reviews, meta-analyses, cohort studies, and guideline-based publications addressing paediatric diagnosis, treatment, survival, neurodevelopment, complications, or quality of life. The review highlights the importance of prenatal ultrasonography, fetal echocardiography, newborn pulse oximetry, echocardiographic confirmation, medical stabilisation, catheter-based procedures, cardiac surgery, intensive care, and structured follow-up. Long-term outcomes have improved, yet residual lesions, arrhythmias, pulmonary hypertension, growth impairment, neurodevelopmental delay, psychosocial burden, and reintervention remain important concerns. Early detection, timely referral, lesion-specific management, and lifelong multidisciplinary surveillance are essential for improving survival, functional status, and quality of life in affected children.

## Introduction and background

Congenital heart diseases (CHDs) encompass a variety of congenital disorders related to structure or function that arise during fetal cardiac development and are evident at birth [1]. CHDs include septal defects, obstructions, cyanotic heart defects, conotruncal defects, valve disorders, and complex single ventricular states [2]. There is marked variability in clinical presentation, ranging from incidental detection during routine examination to critical illness with cyanosis, defined as a bluish discolouration of the skin or mucous membranes caused by inadequate oxygenation, shock, and other life-threatening complications [3]. The most common types of congenital anomalies seen across the world, CHDs, continue to be a significant cause of morbidity, premature death, protracted hospital stays, and excessive healthcare utilisation [4].

Early detection has increasingly emerged as one of the key factors influencing the prognosis in afflicted children. The availability of prenatal ultrasound and fetal echocardiography facilitates early detection of congenital heart defects even before birth, ensuring that infants born with such conditions are delivered in facilities where services for neonatal intensive care, paediatric cardiology, administration of prostaglandins, catheterisation, and cardiac surgical interventions are available [5]. Once born, pulse oximetry screening, physical evaluation, echocardiographic assessment, and referral still represent critical tools for diagnosing congenital abnormalities that may escape detection initially after birth [3]. Delayed diagnosis remains a concern despite these developments, particularly in neonates with subtle clinical findings or duct-dependent lesions, in which adequate blood flow to the lungs or the body temporarily depends on the ductus arteriosus remaining open [6]. Before birth, the ductus arteriosus forms a normal connection between the pulmonary artery and the aorta and allows blood to bypass the fetal lungs. After birth, the lungs expand, and the ductus normally begins to close. In infants with duct-dependent CHD, this closure can abruptly reduce pulmonary or systemic blood flow, resulting in severe oxygen deprivation, poor perfusion, shock, or death [3,6].

As survival has improved, clinical care has expanded beyond correction of the cardiac defect to include recognition and management of anxiety, depression, attention difficulties, behavioural problems, and other mental health disorders that may affect children with CHD [7]. Treatment may include medical stabilisation, management of heart failure and arrhythmias, nutritional support, catheter-based intervention, surgical repair, or staged palliation according to lesion anatomy, physiology, age, weight, comorbidity, and institutional expertise [2,8]. Improved survival has shifted clinical priorities towards long-term outcomes, including ventricular dysfunction, arrhythmias, pulmonary vascular disease, reduced cardiopulmonary fitness, exercise limitation, reintervention, developmental and learning difficulties, psychological burden, and impaired quality of life [6,9,10].

There are important lacunae in the care continuum of paediatric CHD. In India, delayed diagnosis, limited availability of fetal echocardiography, shortages of specialised paediatric cardiac services, financial barriers, and unequal access to surgical treatment continue to affect timely diagnosis and management [11]. Guidelines for neonatal screening, referral, genetic counselling, neurodevelopmental assessment, and transition are not always in place. CHD may adversely affect motor, cognitive, emotional, and social development, particularly among children with complex lesions, chronic hypoxemia, repeated hospitalisation, or major surgical interventions [12]. Other areas of uncertainty include the optimal age for intervention in specific lesions, risk factors for neurodevelopmental deficits, effective and inexpensive screening methods, and follow-up paradigms appropriate for different clinical settings. Evidence from low- and middle-income settings also remains geographically underrepresented, limiting the applicability of findings generated primarily in well-resourced healthcare systems. A clinically oriented review is required to link early diagnosis, treatment modalities, and outcomes in paediatric patients. To maintain accessibility for readers without specialist cardiology training, complex physiological and procedural concepts are introduced in simplified clinical language, whereas detailed diagnostic, pharmacological, catheter-based, and surgical considerations are presented in the relevant later sections.

Objectives of the review

This review aims to analyse current clinical perspectives on early diagnosis, management strategies, and long-term outcomes in children with CHD. It also seeks to identify persistent gaps in screening, referral, multidisciplinary care, neurodevelopmental surveillance, and transition planning across paediatric cardiac practice. This narrative review provides a clinically oriented overview of paediatric CHD, focusing on early detection, principal management approaches, and long-term outcomes; it does not provide a systematic quantitative synthesis or detailed lesion-specific procedural and drug-dosing recommendations.

Methodology

A narrative literature search was conducted using PubMed, Scopus, Web of Science, and Google Scholar to identify publications on early diagnosis, clinical management, and long-term outcomes of CHD in children. The search covered January 2015 to July 2026 and was last updated in July 2026. Selected earlier consensus statements were included when they remained clinically authoritative. The principal search strategy combined terms related to congenital heart disease, paediatric populations, diagnosis, screening, fetal echocardiography, pulse oximetry, medical management, cardiac surgery, interventional cardiology, neurodevelopment, and long-term outcomes. Search terms were adapted to the indexing requirements of each database. Eligible sources included original studies, cohort and observational studies, systematic reviews, meta-analyses, clinical reviews, guidelines, consensus statements, and scientific statements addressing diagnosis, screening, management, complications, survival, neurodevelopment, psychosocial health, or quality of life. Non-English studies, adult-only studies without relevance to lifelong congenital cardiac care, isolated case reports, editorials, conference abstracts without full text, duplicates, and unrelated publications were excluded. Titles and abstracts were screened for relevance, followed by full-text assessment of potentially eligible sources. Extracted information included study design, population, diagnostic or therapeutic focus, principal findings, complications, survival, neurodevelopment, and quality-of-life outcomes. The literature was evaluated for relevance, recency, methodological strength, and citation alignment, then synthesised thematically. As this was a narrative review rather than a systematic review or meta-analysis, PRISMA flow reporting, formal study-level risk-of-bias assessment, and quantitative pooling were not performed. No meta-analysis, meta-regression, effect-size calculation, hypothesis testing, P-values, confidence intervals, or heterogeneity statistics were undertaken because the review did not involve quantitative data pooling; accordingly, additional statistical peer review was not considered necessary.

## Review

Epidemiology and global burden of congenital heart diseases in children

CHD is one of the most commonly observed congenital anomalies and remains a major cause of morbidity and mortality in neonates and children [13]. The reported prevalence varies across regions according to case ascertainment, diagnostic capacity, registry coverage, echocardiographic availability, and infant survival [9,13-15]. Minor lesions, such as small ventricular septal defects (VSDs), may remain undetected during early life, whereas critical congenital heart defects commonly present in neonates with cyanosis, respiratory distress, shock, or heart failure [14].

Geographical variation exists in the epidemiology of CHD, and there tend to be differences in diagnosis among high-income nations and low- and middle-income countries. In high-income nations, CHD cases tend to be diagnosed more frequently due to the use of antenatal ultrasonography, fetal echocardiography, neonatal pulse oximetry, and referrals for paediatric cardiac problems [15]. Improved childhood survival has produced a rapidly growing population of adolescents and adults with CHD, increasing the need for lifelong surveillance, specialised adult congenital cardiac services, and structured transition from paediatric to adult care [16].

Critical lesions that include but are not limited to hypoplastic left heart syndrome, transposition of the great arteries, pulmonary atresia, interrupted aortic arch, and severe coarctation mandate immediate identification and management [13]. Early identification of CHD also enables timely neurodevelopmental risk stratification, longitudinal surveillance, and referral for developmental, educational, psychological, or rehabilitative support when indicated [17]. With improved care in paediatrics, more infants are surviving into childhood, adolescence, and adulthood with either repaired or palliated congenital heart lesions [5]. Thus, care requirements for individuals with congenital cardiac diseases have extended from mere neonatal survival to lifelong monitoring, developmental support, re-interventions, psychological care, and planned transition to adult congenital heart care [14]. Table 1 presents the epidemiological and healthcare burden patterns associated with paediatric CHD.

**Table 1:** Worldwide patterns and healthcare impact of paediatric congenital heart disease. CHD: congenital heart disease; HICs: high-income countries; LMICs: low- and middle-income countries; HF: heart failure; ICU: intensive care unit; POS: pulse oximetry screening; echo: echocardiography.

Epidemiological domain	Global pattern	Main clinical impact	Health-system burden	Citation
Prevalence	Approximately 8-10 CHD cases per 1,000 live births	Early morbidity, variable lesion severity	Need for screening, echo, referral pathways	[13]
Regional variation	Higher detection in HICs, frequent underdiagnosis in LMICs	Delayed diagnosis, advanced presentation	Unequal access to paediatric cardiology and surgery	[15]
Infant mortality	Critical CHD remains a major contributor to neonatal mortality	Cyanosis, shock, HF, sudden deterioration	Emergency stabilisation and urgent transfer needs	[14]
Survival trends	Improved survival after advances in surgery and ICU care	Growing adolescent and adult CHD population	Lifelong follow-up and transition services	[16]
Resource limitations	Limited fetal echo, POS, catheterisation, and surgery in some regions	Preventable deaths and disability	Financial burden, workforce shortage, and delayed intervention	[11]

Classification of congenital heart diseases: A clinically oriented framework

Acyanotic and Cyanotic Lesions

Congenital heart abnormalities can be classified according to oxygenation, haemodynamic effects, anatomical location, and surgical relevance. This framework helps relate cardiac structure to clinical presentation, diagnosis, and treatment [18]. Clinically, congenital heart defects are commonly divided into acyanotic and cyanotic lesions [11]. Acyanotic lesions generally maintain near-normal systemic oxygenation initially, although significant shunting or obstruction may later cause heart failure, pulmonary overcirculation, poor growth, or exercise intolerance [19]. Cyanotic lesions reduce systemic oxygenation through impaired pulmonary blood flow, abnormal mixing, or right-to-left shunting [6].

Left-to-Right Shunt Lesions

Left-to-right shunt lesions include VSD, atrial septal defect (ASD), patent ductus arteriosus (PDA), and atrioventricular septal defect (AVSD). Their clinical significance depends on shunt size and pulmonary blood flow. Haemodynamically important lesions may cause tachypnoea, feeding difficulty, recurrent respiratory infection, poor growth, pulmonary hypertension, and heart failure [20]. Detailed diagnostic and therapeutic considerations are discussed in the subsequent sections.

Obstructive and Duct-Dependent Lesions

Obstructive lesions include pulmonary stenosis, aortic stenosis, coarctation of the aorta, and interrupted aortic arch. Clinical severity ranges from an asymptomatic murmur to circulatory collapse after closure of the ductus arteriosus [13]. Duct-dependent lesions require continued patency of the ductus arteriosus to maintain pulmonary or systemic blood flow. These lesions include hypoplastic left heart syndrome, critical coarctation, pulmonary atresia, severe tetralogy of Fallot, and transposition of the great arteries with inadequate mixing [19]. Delayed recognition may result in severe hypoxemia, metabolic acidosis, shock, or death [19,20].

Single-Ventricle Physiology

Single-ventricle physiology includes tricuspid atresia, double-inlet left ventricle, unbalanced AVSD, and hypoplastic left heart syndrome. These lesions generally require staged palliation, careful physiological management, lifelong surveillance, and transition to adult CHD care [19,20].

Prenatal detection and fetal echocardiography

Identification of CHD in the fetus is an important part of modern fetal medicine. Antenatal ultrasound forms the basis for the identification of anatomical abnormalities of the heart, rhythm disturbances, changes in the dimensions of chambers of the heart, problems with the flow of blood from the heart, and the presence of hydrops or extracardiac congenital anomalies [21]. Normal fetal cardiac scanning involves evaluation of the four-chamber view, outflow tract, three-vessel view, cardiac position, cardiac axis, rhythm, and situs [22]. Fetal echocardiography is especially significant for pregnancies with a heightened risk of CHD. The indications for fetal echocardiography include maternal history of pregestational diabetes mellitus, phenylketonuria, or autoimmune disorders with anti-Ro/SSA or anti-La/SSB antibodies; maternal medication during pregnancy that could cause fetal malformation; assisted reproductive technology; increased nuchal translucency measurement; fetal arrhythmias; extracardiac anomaly; genetic disorder; first-degree relative with CHD; and evidence of cardiac abnormality from prenatal ultrasonography [23]. Associations between genetics and heart disease are clinically useful, since conotruncal abnormalities, AVSD, left-sided obstructions, and heterotaxy syndromes are known to be associated with various chromosomal disorders and gene defects [21].

Prenatal diagnosis does not decrease disease severity; however, it increases readiness. Prenatal identification of serious lesions enables delivery close to the tertiary cardiac care unit, where prostaglandin can be started on time, echocardiography is performed immediately, intensive care admission is planned, and intervention is available through catheterisation or surgery [24]. It facilitates counselling about the outlook, neonatal behaviour, need for staged interventions, chances of neurodevelopmental problems, risk of recurrence, and need for long-term follow-up [20]. The detection rate is still inconsistent among different geographic locations because of disparities in sonographer training, equipment, gestational age at examination, fetal position, mother’s body shape, referral pattern, and lack of paediatric cardiac knowledge [14]. Table 2 summarises the key components and clinical value of prenatal cardiac assessment in suspected paediatric CHD.

**Table 2:** Antenatal cardiac assessment pathways and perinatal implications in paediatric CHD. CHD: congenital heart disease; USG: ultrasonography; Echo: echocardiography; NT: nuchal translucency; CNV: copy number variant; ICU: intensive care unit.

Assessment component	Main indication	Diagnostic contribution	Perinatal care implication	Citation
Routine obstetric USG	Standard antenatal anomaly screening	Identifies abnormal cardiac views, situs, rhythm, hydrops, and extracardiac defects	Triggers referral for fetal echo and specialist review	[24]
Fetal echo	Suspected CHD or high-risk pregnancy	Defines lesion anatomy, physiology, severity, and rhythm status	Guides delivery site, neonatal stabilisation, and intervention planning	[22]
Maternal and family risk review	Diabetes, autoimmune disease, teratogen exposure, and first-degree CHD history	Refines fetal CHD risk and screening priority	Supports targeted imaging and recurrence counselling	[21]
Genetic evaluation	Extracardiac anomaly, increased NT, syndromic features, selected cardiac defects	Detects aneuploidy, CNV, microdeletion, or monogenic association	Informs prognosis, counselling, and multidisciplinary planning	[18]
Perinatal coordination	Critical or duct-dependent CHD	Links fetal cardiology, obstetrics, neonatology, ICU, and surgery teams	Enables planned tertiary delivery and early neonatal intervention	[21,24]

Newborn screening and early postnatal diagnosis

The screening of newborns and assessment in the immediate postnatal period are crucial for the identification of CHD when the clinical manifestations are still vague, occasional, or concealed by transitional circulation [25]. Certain vital abnormalities lack clinical manifestation in the initial hours of life, especially those that depend on the PDA [23]. With the gradual closure of the ductus, the infants will suffer from hypoxemia, metabolic acidosis, respiratory failure, poor perfusion, shock, or acute clinical instability [26]. Pulse oximetry screening has emerged as a useful technique at the bedside to detect critical CHD before discharging the patient from the birthing hospital [27]. Pulse oximetry screening is typically done after the baby has reached at least 24 hours of age to minimise the occurrence of false positives secondary to the physiologic adaptation of the cardiopulmonary system [19]. Preductal saturation measured from the right hand, and postductal saturation assessed from either foot, can demonstrate hypoxemia or abnormal oxygenation that could be indicative of duct-dependent systemic or pulmonary blood flow [15].

The clinical examination is equally essential and should not be substituted by oximetry alone [28]. It involves general appearance, breathing pattern, perfusion, femoral pulses, capillary refill, hepatomegaly, precordial activity, heart sounds, murmurs, and oxygen saturation [26]. The presence of central cyanosis, weak or absent femoral pulses, differential blood pressure, persistent tachypnoea, diaphoresis on feeding, lethargy, poor weight gain, or hepatomegaly suggests severe heart disease [21]. Heart murmurs should be interpreted cautiously because benign transitional murmurs are common in newborns, whereas some critical congenital heart defects may initially occur without an audible murmur [25,28].

**Figure 1:**
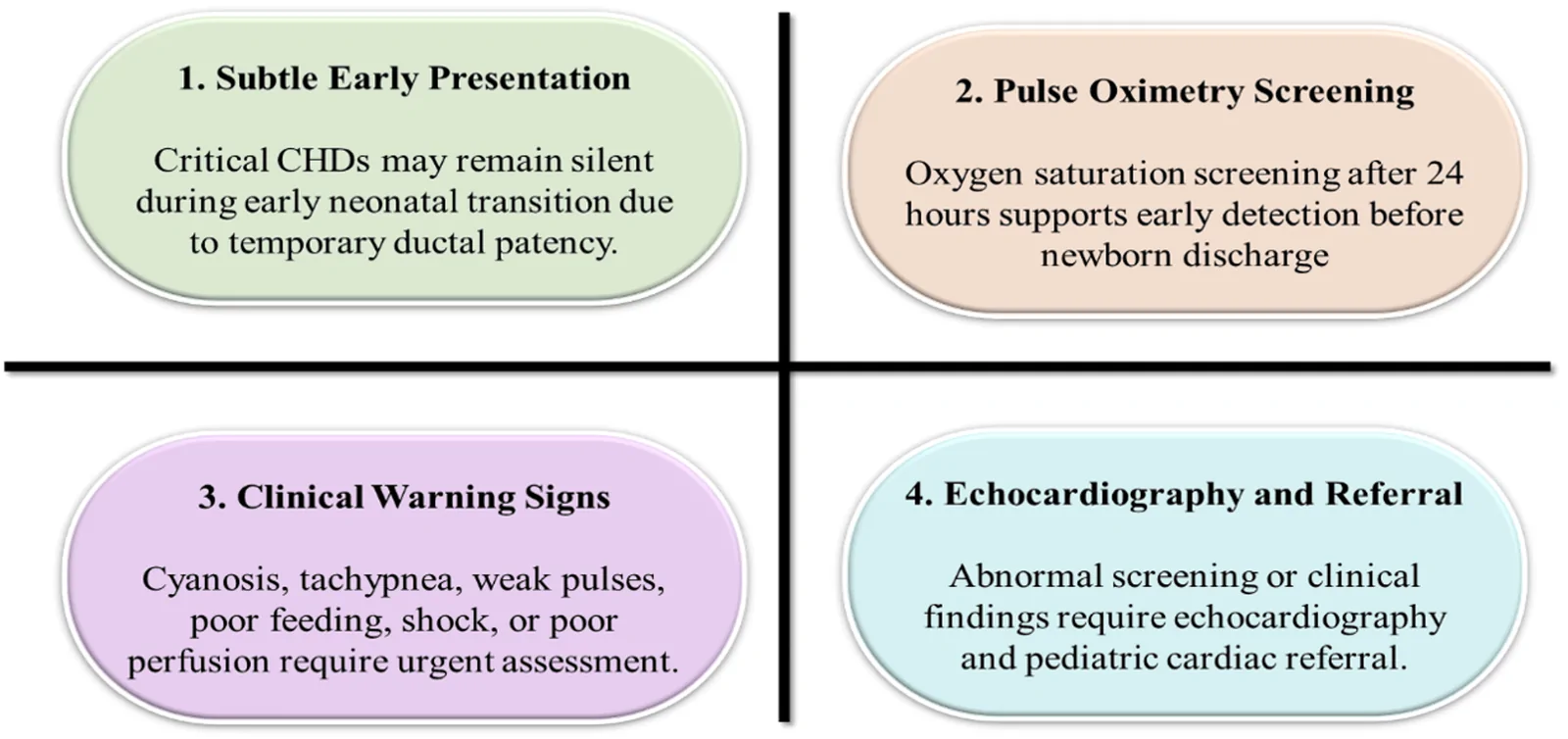
Newborn screening and early diagnosis of paediatric CHD. CHD: congenital heart disease. Created by the authors using Microsoft PowerPoint (Microsoft Corporation, Redmond, WA).

Postnatal diagnosis is also necessary, using an extensive differential diagnosis method. Cyanotic CHD can present similarly to lung diseases, septicaemia, metabolic abnormalities, or persistent pulmonary hypertension [27]. Heart failure associated with a large shunt may appear later in life, usually manifesting as difficulty eating, rapid breathing, recurring respiratory problems, failure to gain weight, or excessive sweating [14]. Obstruction abnormalities like coarctation of the aorta will not appear until after the ductus arteriosus closes and will show as inadequate perfusion, acidosis, kidney problems, or shock [6]. An echocardiogram will be required for diagnosis, assisted by electrocardiograms, chest X-rays, blood gases, lactate levels, and consultations with paediatric cardiologists where possible [24]. Figure 1 shows the major clinical steps involved in early postnatal recognition of paediatric CHD, including subtle presentation, pulse oximetry screening, clinical warning signs, and echocardiographic referral.

Diagnostic modalities in paediatric congenital heart disease

The diagnosis of paediatric CHD requires integration of clinical examination, imaging, physiological assessment, genetics, and selected biomarkers [29].

Echocardiography

Transthoracic echocardiography is the principal diagnostic modality in infants and children because it is widely available, can be performed at the bedside, does not involve ionising radiation, and provides detailed assessment of intracardiac anatomy, ventricular function, valvular abnormalities, shunt direction, pressure gradients, chamber dimensions, and pulmonary arterial anatomy [30]. Doppler echocardiography provides additional haemodynamic information, including the severity of stenosis, valvular regurgitation, pulmonary pressures, and blood-flow characteristics [24]. Its limitations include operator dependence, suboptimal acoustic windows, restricted visualisation of extracardiac structures, and difficulty assessing complex postoperative anatomy [31].

Electrocardiography and Chest Radiography

Electrocardiography provides information on chamber enlargement, axis deviation, conduction abnormalities, ischaemic strain, and arrhythmias but does not define cardiac anatomy [30,32]. Chest radiography may demonstrate cardiomegaly, abnormal pulmonary vascularity, associated lung disease, situs abnormalities, or postoperative device position [29]. These investigations are useful adjuncts but do not replace echocardiography.

Advanced Imaging: Cardiovascular Magnetic Resonance and Computed Tomography Angiography

Cardiovascular magnetic resonance provides a detailed assessment of ventricular volumes and function, myocardial characteristics, blood-flow quantification, pulmonary arteries, systemic veins, the aortic arch, and complex postoperative anatomy [30,33]. It avoids ionising radiation but may be limited by availability, examination duration, the need for sedation or anaesthesia in younger children, and artefacts or safety considerations related to selected implanted devices. Computed tomography angiography offers rapid, high-resolution visualisation of extracardiac vessels, coronary artery anatomy, pulmonary venous connections, airway anatomy, aortic arch abnormalities, and postoperative anatomy, although radiation and contrast exposure must be considered [32].

Diagnostic and Therapeutic Cardiac Catheterisation

Cardiac catheterisation provides direct measurement of intracardiac pressures, oxygen saturations, pulmonary vascular resistance, and angiographic anatomy. It also permits therapeutic procedures such as balloon valvuloplasty, defect closure, angioplasty, and stent placement [22,33]. Potential complications include vascular injury, bleeding, arrhythmia, contrast-associated kidney injury, radiation exposure, and anaesthesia-related adverse events [29,34].

Genetic Evaluation

Genetic evaluation may support syndromic diagnosis, recurrence-risk counselling, prognostication, and screening for associated extracardiac abnormalities, particularly in patients with conotruncal defects, AVSDs, heterotaxy, or left-sided obstructive lesions [30].

Initial stabilisation and medical management

Emergency Assessment and Stabilisation

The initial management of CHD in a neonate or infant involves maintaining adequate systemic perfusion and oxygen delivery, preventing metabolic deterioration, and obtaining timely evaluation by a paediatric cardiologist [34]. Critical CHD may present with cyanosis, respiratory distress, weak pulses, poor feeding, lethargy, metabolic acidosis, hypoglycaemia, oliguria, shock, or a difference between preductal and postductal oxygen saturation [34]. Infants with functionally single-ventricle anatomy require early multidisciplinary planning because staged palliation may ultimately lead to Fontan circulation and lifelong surveillance for multisystem complications [35]. Immediate assessment should include the airway, breathing, circulatory stability, temperature, blood glucose, blood pressure in all extremities, preductal and postductal oxygen saturation, peripheral perfusion, serum lactate, acid-base status, and echocardiographic findings [27,34].

Maintenance of Ductal Patency

Prostaglandin E1 should be initiated promptly when duct-dependent CHD is suspected to maintain ductal patency until definitive catheter-based or surgical intervention can be performed [34,36]. It is indicated when pulmonary or systemic blood flow depends on the ductus arteriosus, including pulmonary atresia, critical pulmonary stenosis, severe tetralogy of Fallot, hypoplastic left heart syndrome, interrupted aortic arch, and critical coarctation of the aorta [26,34]. Potential adverse effects include apnoea, hypotension, fever, flushing, and electrolyte abnormalities; continuous cardiorespiratory monitoring and immediate access to ventilatory support are therefore required [34,37]. Oxygen should be administered cautiously because excessive oxygenation may reduce pulmonary vascular resistance and increase pulmonary overcirculation in selected patients with mixing lesions or single-ventricle physiology [21,34].

Heart Failure and Circulatory Support

Medical management of heart failure may include diuretics, careful fluid management, afterload reduction, nutritional support, and treatment of contributing conditions such as anaemia or infection [31,37]. Diuretics may reduce pulmonary congestion in children with large left-to-right shunts, atrioventricular valve regurgitation, or ventricular dysfunction [31]. Afterload reduction may improve systemic output in selected patients with ventricular dysfunction or substantial regurgitant lesions [31,38]. Vasoactive or inotropic agents, including dopamine, dobutamine, epinephrine, milrinone, or norepinephrine, may be required in patients with shock, low cardiac output, or postoperative haemodynamic instability [34,37]. Arrhythmia management should include identification and correction of reversible factors such as hypoxemia, acidosis, electrolyte abnormalities, medication toxicity, fever, and haemodynamic instability [29,38].

Nutrition and Infection Prevention

Nutritional support is an important component of management because children with significant congenital heart defects may have increased metabolic requirements, feeding fatigue, poor weight gain, and an increased risk of aspiration [34,38]. Calorie-dense feeding, nasogastric supplementation, breastfeeding support, and dietitian-directed care may improve preoperative and postoperative growth [34]. Infection-prevention measures include age-appropriate immunisation, hand hygiene, prompt assessment and treatment of respiratory infections, appropriate perioperative antibiotic prophylaxis, maintenance of oral health, and infective endocarditis prophylaxis for selected high-risk cardiac conditions and procedures [29,32]. Table 3 summarises the principal stabilisation and medical-management strategies used in critically ill infants with CHD.

**Table 3:** Acute supportive care measures and therapeutic priorities in paediatric CHD. CHD: congenital heart disease; PGE1: prostaglandin E1; BP: blood pressure; SpO₂: peripheral oxygen saturation; ABG: arterial blood gas; LCOS: low cardiac output syndrome; HF: heart failure; NG: nasogastric.

Management domain	Main intervention	Clinical indication	Monitoring priority	Citation
Ductal patency	PGE1 infusion	Duct-dependent pulmonary or systemic circulation	Apnoea, BP, temperature, perfusion, lactate	[37]
Oxygen strategy	Controlled oxygen therapy	Cyanosis, hypoxemia, respiratory distress	Preductal and postductal SpO₂, ABG, systemic perfusion	[34]
Circulatory support	Inotropes and vasoactive agents	Shock, LCOS, ventricular dysfunction	BP, urine output, lactate, rhythm, capillary refill	[31]
HF management	Diuretics, fluid control, afterload reduction	Pulmonary congestion, large shunt, ventricular dysfunction	Electrolytes, renal function, weight, and respiratory status	[32]
Growth and prevention	Calorie-dense feeds, NG support, infection prevention	Feeding fatigue, poor weight gain, high-risk cardiac physiology	Weight gain, intake volume, aspiration risk, and fever signs	[36]

Interventional cardiology and surgical management

Interventional cardiology and cardiac surgery provide definitive treatment for many children with CHD. The choice of catheter-based, surgical, or combined intervention depends on cardiac anatomy, physiology, age, weight, ventricular function, pulmonary artery anatomy, genetic or extracardiac conditions, and institutional expertise [39].

Catheter-Based Interventions

Catheter-based treatment is increasingly used for selected obstructive lesions, intracardiac communications, and vascular abnormalities, often reducing the need for open surgery and shortening recovery [39]. In selected children with trisomy 13 or 18, CHD intervention may be considered after individualised assessment of cardiac anatomy, associated conditions, expected clinical benefit, and family preferences [40]. Balloon valvuloplasty is an established treatment for valvar pulmonary stenosis and selected cases of congenital aortic stenosis [41]. Balloon angioplasty or stent placement may be used for coarctation of the aorta, branch pulmonary artery stenosis, ductal obstruction, or postoperative vascular restenosis [36]. Transcatheter closure may be considered for anatomically suitable ASDs, PDA, selected muscular VSDs, and residual postoperative communications [42]. Transcatheter pulmonary valve replacement may be used in selected children and young adults with right ventricular outflow tract dysfunction after previous repair [38,41]. Access to paediatric catheterisation and cardiac surgery remains limited in many low- and lower-middle-income countries, contributing to delayed intervention and preventable adverse outcomes [43]. Catheter-based procedures require detailed preprocedural anatomical and physiological assessment and may be complicated by vascular injury, embolisation, arrhythmia, residual obstruction, device malposition, valvular regurgitation, contrast exposure, or ionising radiation [29,34].

Surgical Repair

Surgery remains essential for complex CHD, large defects unsuitable for transcatheter closure, cyanotic lesions, aortic arch obstruction, anomalous vascular connections, and functionally single-ventricle anatomy [29,38,42]. Complete repair may be achieved for lesions such as VSD, AVSD, tetralogy of Fallot, transposition of the great arteries, total anomalous pulmonary venous connection, and selected forms of truncus arteriosus [37].

Staged Palliation for Single-Ventricle Physiology

Complex lesions that cannot undergo biventricular repair may require staged palliation. Children with functionally single-ventricle anatomy commonly undergo a neonatal systemic-to-pulmonary shunt or Norwood-type procedure, followed by a bidirectional Glenn anastomosis and subsequent Fontan completion [35,42].

Perioperative Risks and Reintervention

**Figure 2:**
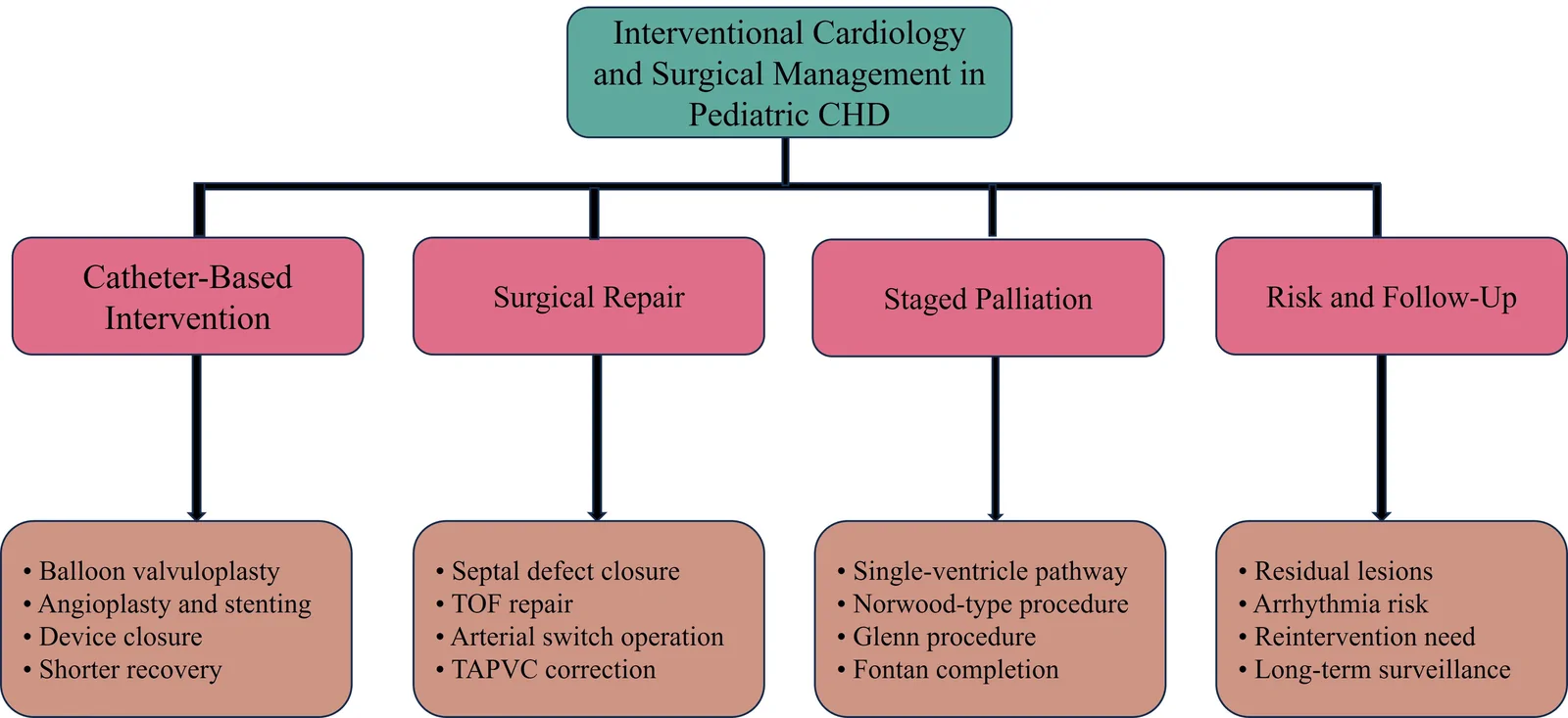
Interventional and surgical treatment pathways for paediatric CHD. CHD: congenital heart disease. Created by the authors using Microsoft PowerPoint (Microsoft Corporation, Redmond, WA).

Perioperative risk is influenced by prematurity, low birth weight, cyanosis, ventricular dysfunction, pulmonary hypertension, renal impairment, infection, genetic abnormalities, extracardiac disease, and procedural complexity [20,44]. Postoperative complications may include low cardiac output syndrome, arrhythmias, bleeding, residual lesions, pleural effusion, chylothorax, pulmonary hypertensive crises, infection, neurological complications, and the need for catheter-based or surgical reintervention [27,44]. Long-term success depends on continued surveillance of residual haemodynamic abnormalities, ventricular function, growth, exercise capacity, neurodevelopment, and quality of life [38,44]. Figure 2 illustrates the principal catheter-based, surgical, and staged-palliation pathways used in paediatric CHD.

Postoperative care, complications, and multidisciplinary follow-up

Postoperative management for CHD involves constant evaluation of the following parameters: haemodynamic stability, oxygenation, ventilation, tissue perfusion, rhythm, fluid volume, and end-organ function [44]. Before discharge, parents and caregivers require structured education regarding medication administration, feeding, wound care, warning signs, activity restrictions, follow-up appointments, and circumstances requiring urgent medical assessment [45]. Postoperative monitoring may include arterial blood pressure, central venous pressure, blood gases, serum lactate, urine output, electrocardiography, echocardiography, coagulation indices, renal function, infection markers, and, in selected patients, near-infrared spectroscopy [44,46,47].

The need for postoperative rehabilitation is influenced by defect complexity, age, neurological or developmental vulnerability, prolonged hospitalisation, and postoperative functional impairment [46]. Neonates with CHD may demonstrate abnormal cerebral haemodynamics, and cerebral perfusion can change following surgical intervention, supporting careful neurological and developmental surveillance [47]. Arrhythmias occur commonly after the correction of atrial, ventricular, or outflow tract defects and can be manifested by junctional ectopic tachycardia, supraventricular tachycardia, atrioventricular block, atrial flutter, and ventricular tachyarrhythmia [44]. Age-appropriate health education and empowerment-based support may improve children’s understanding of surgery, participation in care, coping ability, and postoperative adjustment [48].

Anticoagulant or antiplatelet therapy may be required after prosthetic valve implantation, systemic-to-pulmonary shunt placement, Fontan palliation, vascular stenting, or in patients with specified thrombotic risk factors [35,38]. Antithrombotic therapy requires an individualised balance between thrombosis prevention and bleeding risk, with attention to growth-related dose adjustment, adherence, and laboratory or clinical monitoring [35,38]. Prevention of infective endocarditis includes maintenance of oral hygiene, patient and parent education, prompt evaluation of suspected infection, and antibiotic prophylaxis for selected high-risk cardiac conditions and procedures [29,32]. Recovery is not only about cardiovascular stability. There can be problems like eating troubles, raised metabolic needs, malfunctioning of vocal cords, danger of aspirating food into the lungs, and extended stays in the hospital that affect normal growth [39]. Nutrition programs based on dietitian recommendations, feeding therapy, physical therapy, developmental screening, and psychological counselling can help with functional recovery [28]. Follow-up care requires coordination between paediatric cardiology, cardiac surgery, intensive care units, general paediatrics, nursing, nutrition, physical therapy, psychology, and development.

Long-term outcomes: Survival, neurodevelopment, quality of life, and psychosocial health

The long-term outlook for children with congenital heart defects has greatly improved because of improvements in prenatal diagnosis, neonatal resuscitation, paediatric cardiothoracic surgery, interventional catheterisation, anaesthesiology, critical care medicine, and long-term monitoring [49]. Life expectancy through adolescence and adulthood can be expected for many conditions that formerly had high perinatal mortality rates [28]. Improved childhood survival has produced a rapidly growing population of adolescents and adults with CHD, increasing the need for lifelong surveillance, specialised adult congenital cardiac services, and structured transition from paediatric to adult care [16]. Residual morbidity is prevalent following repair or palliation. Children and adults with Fontan circulation require lifelong multidisciplinary surveillance for ventricular dysfunction, arrhythmias, thromboembolism, exercise intolerance, protein-losing enteropathy, plastic bronchitis, and hepatic, renal, and lymphatic complications [35]. Children may experience ventricular dysfunction, arrhythmias, pulmonary hypertension, residual shunting, valvular abnormalities, cyanosis, thromboembolic complications, reduced exercise capacity, recurrent hospitalisation, and the need for catheter-based or surgical reintervention [35,38,44]. Complex CHD, single-ventricle physiology, prematurity, genetic conditions, chronic or perioperative hypoxia, prolonged intensive-care treatment, and low cardiac output increase the risk of adverse long-term outcomes [17,44,46,47]. Periodic cardiological assessment, radiological evaluation, rhythm analysis, exercise testing, and drug management are necessary for early detection of deterioration before permanent damage occurs [39].

Neurodevelopmental status is a critical determinant of long-term functioning and quality of life. Children with CHD, particularly those with complex lesions or recognised clinical risk factors, may experience motor delay, language impairment, executive-function difficulties, attention problems, learning disabilities, and reduced academic achievement [17,49]. Risk is influenced by altered fetal brain development, impaired cerebral oxygen delivery, genetic or extracardiac abnormalities, prematurity, perioperative neurological injury, seizures, prolonged hospitalisation, and socioeconomic disadvantage [17,47,49]. Symptom prevalence, physical limitations, invasive procedures, medication dependency, visibility of surgical scars, time away from school, involvement with peers, and concerns about future health problems are all contributors to the quality of life for people with CHD [49]. Children and adolescents with CHD may experience anxiety, depression, attention difficulties, behavioural problems, sleep disturbance, and reduced self-esteem [7,49]. The effect on families may also be substantial, including psychological stress, financial strain, disruption of daily routines, and persistent concern about the child’s health [5].

Limitations and future recommendations

This review is limited by the narrative method of organising information, where selection bias could occur, and comparison across studies becomes limited quantitatively. Differences between patient groups, extent of brain injury, diagnosis criteria, length of follow-up, level of surgical skill, and health infrastructure could influence the interpretation of results. Studies from low- and middle-income countries are underrepresented in the available literature. Long-term effects such as development, psychosocial issues, academic performance, and transition skills have not been consistently evaluated.

Further research must focus on a multicentre prospective design with standardised criteria for diagnosis, common outcomes, and extended follow-ups into adulthood. There must be an increased focus on prenatal detection, neonatal screening, early referrals, neurodevelopmental monitoring, family-based interventions, and transition services. More cost-efficient systems for resource-poor environments must be created, which will be possible through registries, telemedicine, staff training, and accessibility to paediatric cardiac surgery and interventional cardiology.

## Conclusions

Heart diseases among children represent a significant problem clinically and public health-wise, with the results being highly dependent on early diagnosis, prompt stabilisation, appropriate treatment according to specific lesions, and continuous multidisciplinary management. Improvements in fetal echocardiography, pulse oximetry screening in newborns, paediatric cardiology imaging techniques, catheterisation procedures, cardiac surgeries, intensive care, and perioperative management have increased the survival chances of children with many simple and complex heart diseases. Continuous management will always be important since repaired or palliated patients may still develop other complications. Management necessitates collaboration among disciplines such as paediatric cardiology, neonatology, cardiac surgery, intensive care, genetics, nutrition, physiotherapy, developmental medicine, psychology, and general practice. Ongoing disparities exist in areas such as fetal diagnosis, specialist consultation, surgery availability, and long-term surveillance, which significantly impact outcomes. Enhancing processes of screening, referral, counselling, neurodevelopmental tracking, and transitioning to adulthood can contribute positively towards improving survival rates, physical function, quality of life, and long-term cardiovascular health of patients with CHD.
